# Mapping Quantitative Trait Loci Affecting *Arabidopsis thaliana* Seed Morphology Features Extracted Computationally From Images

**DOI:** 10.1534/g3.112.003806

**Published:** 2013-01-01

**Authors:** Candace R. Moore, David S. Gronwall, Nathan D. Miller, Edgar P. Spalding

**Affiliations:** Department of Botany, University of Wisconsin, Madison, Wisconsin 53706

**Keywords:** seed QTL, seed size, image processing, embryo morphology

## Abstract

Seeds are studied to understand dispersal and establishment of the next generation, as units of agricultural yield, and for other important reasons. Thus, elucidating the genetic architecture of seed size and shape traits will benefit basic and applied plant biology research. This study sought quantitative trait loci (QTL) controlling the size and shape of *Arabidopsis thaliana* seeds by computational analysis of seed phenotypes in recombinant inbred lines derived from the small-seeded Landsberg *erecta* × large-seeded Cape Verde Islands accessions. On the order of 10^3^ seeds from each recombinant inbred line were automatically measured with flatbed photo scanners and custom image analysis software. The eight significant QTL affecting seed area explained 63% of the variation, and overlapped with five of the six major-axis (length) QTL and three of the five minor-axis (width) QTL, which accounted for 57% and 38% of the variation in those traits, respectively. Because the Arabidopsis seed is exalbuminous, lacking an endosperm at maturity, the results are relatable to embryo length and width. The Cvi allele generally had a positive effect of 2.6–4.0%. Analysis of variance showed heritability of the three traits ranged between 60% and 73%. Repeating the experiment with 2.2 million seeds from a separate harvest of the RIL population and approximately 0.5 million seeds from 92 near-isogenic lines confirmed the aforementioned results. Structured for download are files containing phenotype measurements, all sets of seed images, and the seed trait measuring tool.

The seed disperses, protects, and sustains the beginning stage of the next generation in plants that produce them. Postgermination survival and subsequent development of the next generation depends on seed parameters such as size (Krannitz *et al.* 1991; Manning *et al.* 2009). Also, the seed is the primary product of many agricultural crops, and biotechnologists are endeavoring to engineer its chemical composition and properties (Abelson & Hines 1999; Ufaz & Galili 2008). Thus, an important goal in basic and applied plant biology is to elucidate the genetic elements responsible for controlling seed size and shape. Quantitative trait locus (QTL) analysis based on phenotypic data from many plants harboring different and known combinations of two distinct parental DNA types is a proven approach to this goal (Alonso-Blanco *et al.* 2009). Many excellent populations of such recombinant inbred lines (RILs) have been created in *Arabidopsis thaliana* because its short generation time, natural self-pollination trait, and numerous diverse accessions are conducive to their generation (Meyerowitz 2001; Kover & Mott 2012). For example, the collection of 162 Arabidopsis RILs derived from a cross between the Landsberg *erecta* (L*er*) and Cape Verde Islands (Cvi) accessions supports mapping of QTL to genomic intervals smaller than a cM (Alonso-Blanco *et al.* 1998). Thus, the germplasm and genotype information are strengths of Arabidopsis QTL studies. What typically limits the quality of such studies is the quality of the phenotype dataset. Traits can be difficult to measure repeatedly and precisely in the many members of the RIL population. In the case of seeds, this is especially true because each elliptical Arabidopsis seed containing the next generation in embryonic form is less than a millimeter long. A pioneering seed size QTL study was previously performed with the Cvi × L*er* RIL population (Alonso-Blanco *et al.* 1999) in which seed size was measured by microscope-aided inspection. The limitations in throughput and precision inherent in such a manual technique for small object measurement can be alleviated by using a flatbed photo scanner to acquire digital images containing many seeds in a field of view, and image processing techniques to quantify features such as the projected area of each separate seed (Herridge *et al.* 2011; Schneider *et al.* 2012). The project reported here represents an extension of the growing trend to use computational methodologies for precision phenotype measurements and the especially robust genotype-to-phenotype mapping that this approach enables. In addition to reporting the genetic architecture of the morphology of Arabidopsis seeds and therefore of the embryo plant within, the present report makes available to the community a comprehensive image set (the raw data), the quantified morphological features of many thousands of seeds from the Cvi × L*er* population (the processed trait data), and a software tool for creating the latter from the former.

## Materials and Methods

### Plant material

This study used the 162 genotyped RILs derived from the L*er*- and Cvi-inbred parents (Alonso-Blanco *et al.* 1999). The seeds used in the RIL1 population were kindly provided by Dr. Patrick Masson from the University of Wisconsin-Madison, Madison, WI. The exact growth conditions of this population are unknown. RIL2 data were collected from a second harvest of seeds produced by six replicate plants for each RIL grown in a randomized pattern in an air-conditioned greenhouse environment at the University of Wisconsin-Madison Biotron. Temperature was maintained at 23° during the 16-hr day and 21° during the night. The beds were watered with 0.25× Hoagland’s solution twice a week for the first month, followed by once a week for the remaining growth period. At appearance of the first flower, each pot was self-contained using ArabiSifters (SNS-02, Lehle Seeds, Round Rock, TX) to prevent cross-pollination. At maturity, each plant was dried and its seeds were sifted through a coarse mesh to remove plant debris before being placed in a plastic tube for storage. A set of 92 NILs was obtained through ABRC. Seeds from each NIL were produced and harvested as described previously.

### Seed trait measurements

Approximately 1000 seeds from each mother plant were sprinkled onto a square Petri dish, and scanned using an Epson Perfection 4990 Photo series scanner to obtain an 8-bit grayscale image. RIL populations were scanned at 3200 dots per inch (dpi), and the NIL population was scanned at 4800 dpi. A custom computer program was developed to detect seeds in the images thus produced and measured the seed area, seed length (major axis), and seed width (minor axis) for each detected seed. To summarize, the tasks performed by the algorithm for extracting and measuring the seeds present in an image are:

- obtain and store a threshold value that segments the grayscale image into foreground (seeds) and background;
- identify each potential seed in the binarized image and determine its area, minor axis and major axis; and
- filter results to reject image components not corresponding to individual seeds.

Task 1 is a multistep process because variation in the background values and the number of seeds present produces variation in the histograms used to select the most effective threshold value by a standard method (Otsu 1979). No single threshold value is optimal for all images, and if an image contains very few seeds, and therefore a low number of black pixels, the Otsu method may not produce a useful result. To obtain a threshold value suitable for all images in a batch, the optimal threshold value for each individual image is determined. These individual values are averaged, and the mean threshold is used to binarize all images in the batch. Task 2 identifies each component of the image consisting of 8-connected black pixels, the potential seeds, and computes the area, major axis, and minor axis of each of these 8-connected components. The results are saved to disk. Task 3 determines which of the identified and measured components has the characteristics of an ellipsoidal seed. From the major and minor axes determined for each 8-connected component, the area is calculated assuming the object is ellipsoidal. This modeled area is compared with the measured area. If the two agree, the component passes the first stage of filtering, which effectively removes instances of two touching seeds and some debris. The second filtering step removes components that pass the ellipse test but which have a major axis more than 2.5 times longer than the minor axis, and therefore are not seeds. A scratch in the Petri plate is an example of an artifact this heuristic was implemented to remove. The area, major axis, and minor axis derived from image components after filtering to remove nonseeds were the traits subjected to statistical genetic analyses.

The algorithm, written in the Matlab language and operating instructions is presented for download at http://phytomorph.wisc.edu/G3. Also presented there for download are all the raw images and the comma separated value files containing the filtered results obtained from each image.

### QTL analysis

After image analysis, the mean seed area, major axis, and minor axis were computed for each set of progeny. For the RIL2 and NIL populations, the final phenotype value for each line is the average of all the replicates. Genotype information from 234 markers in the L*er*/Cvi RIL map and 102 markers from the L*er*/Cvi NIL map was collected from previously published work (Alonso-Blanco *et al.* 1998; Keurentjes *et al.* 2007). The qtl library (Broman *et al.* 2003) within the R statistical software (www.r-project.org/) was used to search for and characterize significant loci linked to the markers using multiple-QTL mapping methods. Two hundred and fifty-six rounds of imputation (Sen & Churchill 2001) were performed using pseudomarkers at 1-cM intervals. A genotyping error rate of 0.001 was assumed, and the Kosambi map function (Kosambi 1944) was used to estimate genetic distances. Significance thresholds (α = 0.05) were calculated by a permutation test (Churchill & Doerge 1994) of a two-dimensional, two-QTL scan analyzed by Haley-Knott regression (Haley & Knott 1992) using 25,000 permutation replicates. Applying significance thresholds calculated in this manner produced essentially the same results as those obtained by the imputation method. The best QTL model was selected using the stepwise QTL analysis of Manichaikul *et al.* (2009) with model selection proceeding up to 10 QTL. This approach seeks as many true QTL as possible, minimizes the inclusion of extraneous loci, but is permissive of extraneous interactions joining the model as the number of potentially interacting QTL increases.

Forward and backward searches of QTL models were performed. The quality of each potential model was evaluated using a penalized logarithm of odds (LOD) score that balances model fit and model complexity by subtracting a penalty derived from the permutation tests for each additional QTL or QTL:QTL interaction present in the model. The LOD penalties were calculated for main effect QTL and epistatic interactions on the basis of the thresholds derived from the scantwo permutation tests. The main, heavy, and light interaction penalties as well as the 5% significance thresholds are presented in accordance with Broman and Sen (2009) in the appropriate table legend. The models generated by the stepwise QTL method of Manichaikul *et al.* (2009) include only QTL and interactions deemed to be significant using the permutation thresholds previously calculated. The chosen model is the one which has the highest penalized LOD score among all models evaluated. Positions of QTL in the final model were refined, then fit to the phenotypic data to provide estimates of each QTL’s effect and LOD score from the fit of the full model. A 1.5 LOD support interval was used to determine the confidence intervals for each locus.

## Results

**Figure 1:**
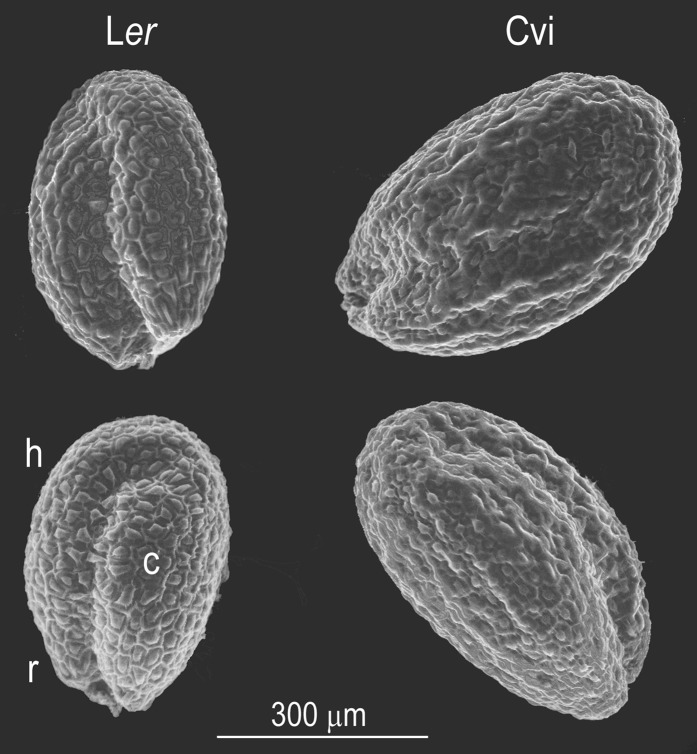
Scanning electron micrographs of two seeds from the L*er* and two seeds of the Cvi accession. c, cotyledon; h, hypocotyl; r, embryonic root or radicle. Scale bar = 300 μm.

Figure 1 shows scanning electron micrographs of two representative seeds from each of the parental accessions used to create the RILs studied here. The images show a typical size difference between Cvi and L*er* seeds and convey how the embryo folded within determines the size and shape of the exalbuminous seed (Esau 1953). It is clear from these images that the length (major axis) reflects the length of the hypocotyl−root axis and the width (minor axis) is essentially the sum of the cotyledon and hypocotyl widths. Thus, a high-resolution quantification of Arabidopsis seed morphology will relate to embryo structure to a considerable extent.

**Figure 2:**
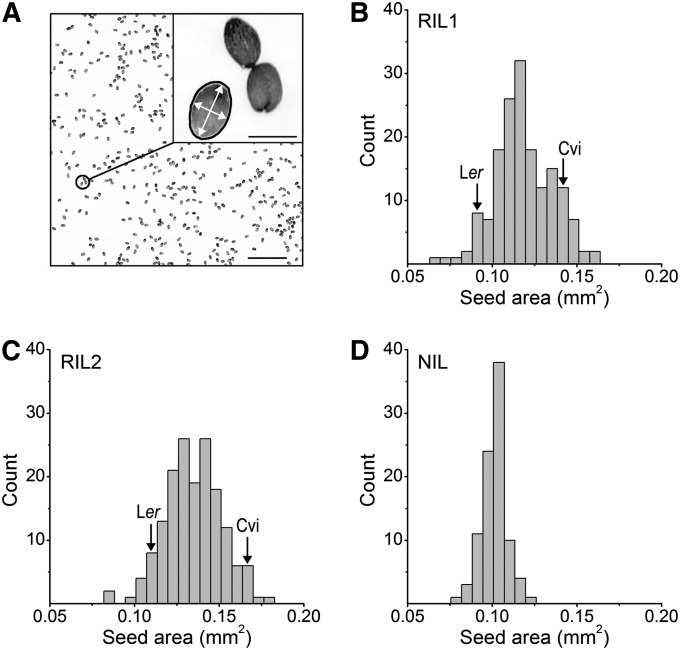
Arabidopsis seed area and shape measured by automatic image processing. (A) A sample of a typical image of a field of Arabidopsis seeds acquired with a flatbed photo scanner. Scale bar = 5 mm. (Inset) An expansion of the field showing a cluster of three seeds. A custom algorithm determines the boundary, or contour (black line), of all objects in the image that can be separated (segmented) from the background and rejects any not having the morphological properties of a single seed such as the two touching seeds shown, or a piece of debris. The white arrows indicate the major and minor axes of a successfully segmented individual seed. Scale bar = 0.5 mm. (B−D), Frequency distribution of seed area in two independent generations of the Cvi × L*er* RIL population (B, C) and a Cvi × L*er* NIL population (D). The 95% confidence interval of the parental means lies within the specified bins.

The scanning electron microscope captures exquisite structural details of the Arabidopsis seed but the imaging procedures cannot be performed with sufficient throughput and automation to support a statistical study of the QTL affecting seed morphology. An appropriate combination of resolution and throughput was achieved with a flatbed photo scanner set to acquire images of seeds scattered in a clear dish at 3200 dpi. Figure 2 shows that the seeds in each of the resulting images were resolved well enough that a computer algorithm could be coded to determine the pixels comprising the contours of each individual seed and reject instances of two or more touching seeds (Figure 2A *inset*). The algorithm automatically returned the area, major axis length, and minor axis length of each successfully segmented, individual seed. On average, 1600 seeds were measured per image. The algorithm in the form of two Matlab code files is presented for download at http://phytomorph.wisc.edu/G3.

The Cvi × L*er* population consists of 162 distinct RILs (Alonso-Blanco *et al.* 1998), but RIL #157 was not included in this study. Aliquots of seeds from each of the remaining 161 RILs were separately scanned and the images processed to produce a dataset containing the average area, major axis length, and minor axis length for each RIL. This dataset is referred to hereafter as RIL1. Figure 2B shows the frequency distribution of the area trait within RIL1. The Cvi and L*er* parental means are indicated to show the extent of transgressive segregation in the population. A separate harvest of this population of RILs was generated and aliquots of their seeds scanned and measured to create the RIL2 dataset. Figure 2C shows the frequency of the seed area trait within RIL2. Near-isogenic lines (NILs) frequently are helpful in detecting small-effect QTL. Therefore, 92 unique NILs each containing one small genomic region of Cvi introgressed into L*er* (Keurentjes *et al.* 2007) were raised to produce seeds that were scanned at 4800 dpi and measured by the same technique. Figure 2D shows the frequency of the seed area trait within the resulting NIL dataset.

Analysis of variance was performed to determine the heritability (*H^2^*) of the traits in each of the datasets (Table 1). For RIL1 and RIL2, *H^2^* ranged from a low of 52% (RIL2 minor axis) to a high of 73% (RIL1 area). Heritability in NIL was much lower due to the limited genetic variation between introgression lines.

**Table 1:** Heritability for seed shape traits estimated from analysis of variance

Trait	MS_M_	MS_E_	df_M_	df_E_	V_A_	V_E_	*H*^2^
RIL1							
Area	0.266	2.28 × 10^−4^	160	1.64 × 10^5^	2.60 × 10^−4^	9.78 × 10^−5^	0.727
Major axis	1.54	1.52 × 10^−3^	160	1.64 × 10^5^	1.50 × 10^−3^	7.68 × 10^−4^	0.662
Minor axis	0.421	4.79 × 10^−4^	160	1.64 × 10^5^	4.12 × 10^−4^	2.73 × 10^−4^	0.601
RIL2							
Area	3.61	2.84 × 10^−4^	161	2.18 × 10^6^	2.69 × 10^−4^	1.50 × 10^−4^	0.642
Major axis	17.3	1.57 × 10^−3^	161	2.18 × 10^6^	1.29 × 10^−3^	9.25 × 10^−4^	0.582
Minor axis	5.93	6.26 × 10^−4^	161	2.18 × 10^6^	4.41 × 10^−4^	4.05 × 10^−4^	0.521
NIL							
Area	0.265	2.24 × 10^−4^	91	4.77 × 10^5^	5.10 × 10^−5^	1.98 × 10^−4^	0.205
Major axis	2.28	1.79 × 10^−3^	91	4.77 × 10^5^	4.40 × 10^−4^	1.57 × 10^−3^	0.219
Minor axis	0.403	6.02 × 10^−4^	91	4.77 × 10^5^	7.76 × 10^−5^	5.63 × 10^−4^	0.121

MS_M_, mean square model; MS_E_, mean square error; d_M_, degrees of freedom of model; df_E_, degrees of freedom of error, V_A_, additive genetic variance; V_E_, environmental variance; *H*^2^, heritability.

**Figure 3:**
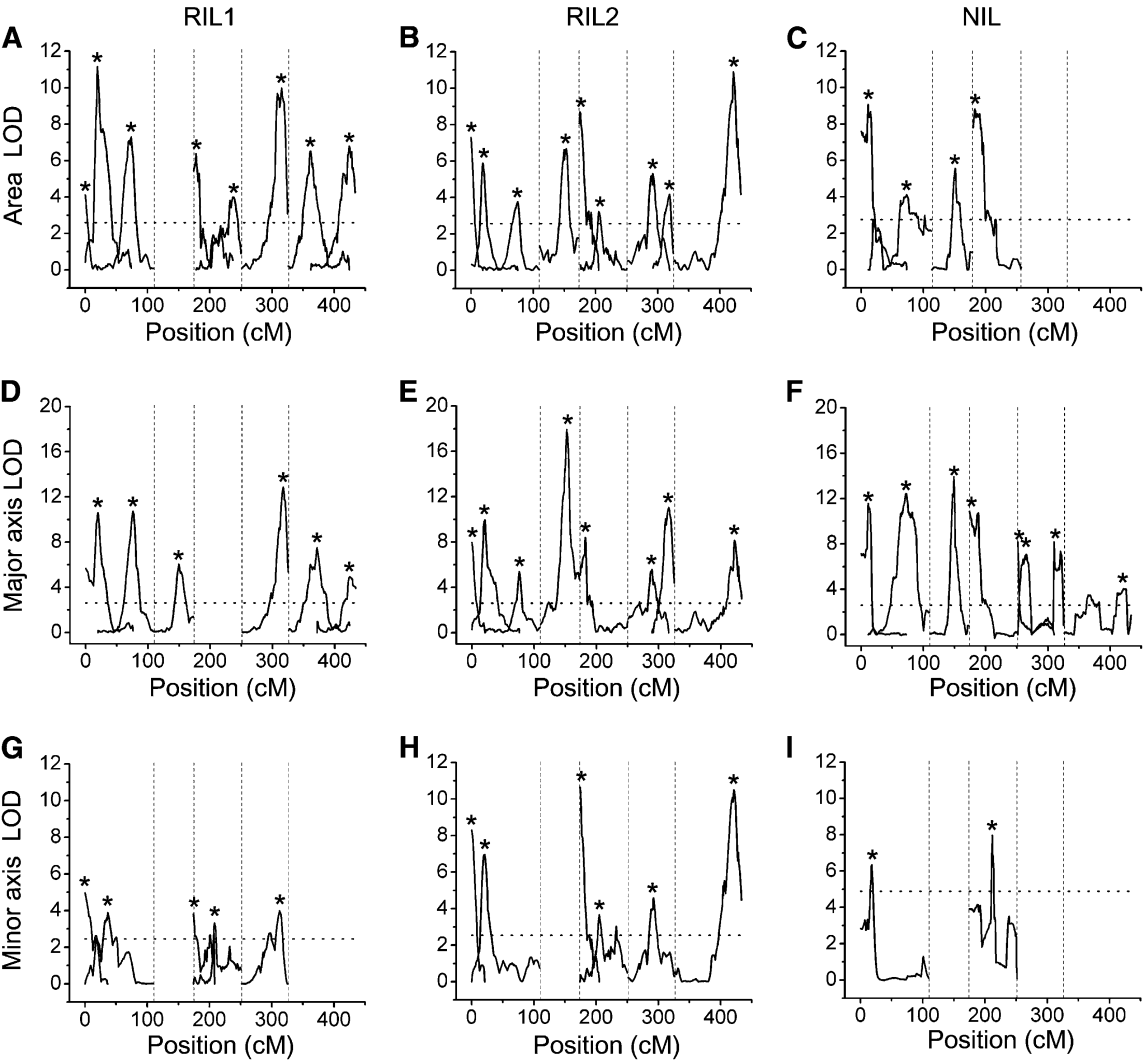
LOD profiles of seed traits statistically modeled as a function of genotype. (A−C), Seed area QTL determined by multiple-interval mapping using the two independent RIL datasets (A, B) and the NIL dataset (C). (D−F), Major axis QTL of the RIL datasets (D, E) and the NIL dataset (F). (G−I), Minor axis QTL of the RIL datasets (G, H), and the NIL dataset (I). Asterisks denote the position with the highest LOD score for each locus. Vertical dotted lines are used to separate the five chromosomes. Horizontal, dotted lines indicates the significance threshold.

**Figure 4:**
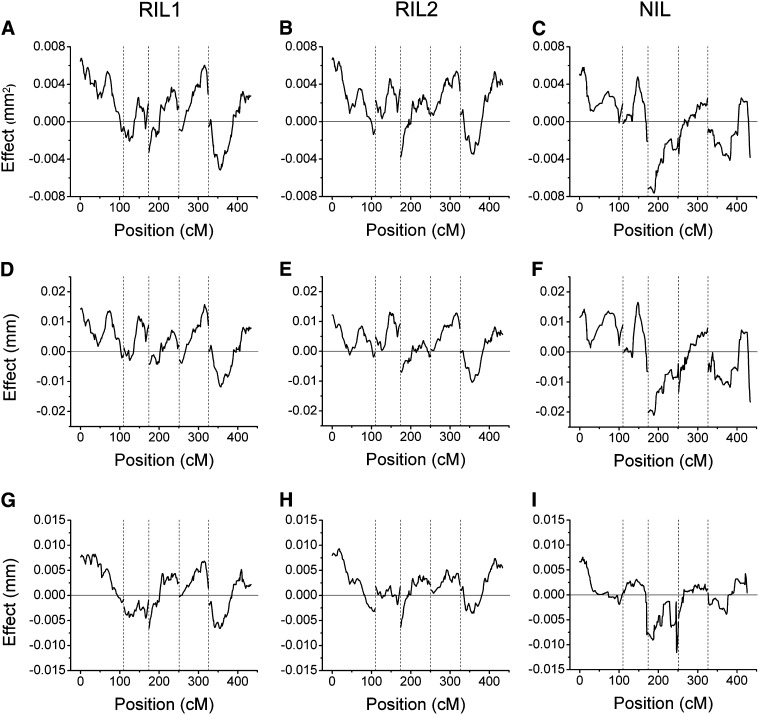
Effects on the seed area trait of each marked position of the genome determined from the indicated dataset. Positive values indicate that substitution of a Cvi allele increases the trait; negative values indicate a L*er* allele at that position increases the trait in the indicated dataset. (A−C), Allele effects on seed area determined from the two independent RIL datasets (A, B) and the NIL dataset (C). (D−F), Allele effects on major axis QTL of the RIL datasets (D, E) and the NIL dataset (F). (G−I), Allele effects on minor axis QTL of the RIL datasets (G, H) and the NIL dataset (I).

**Figure 5:**
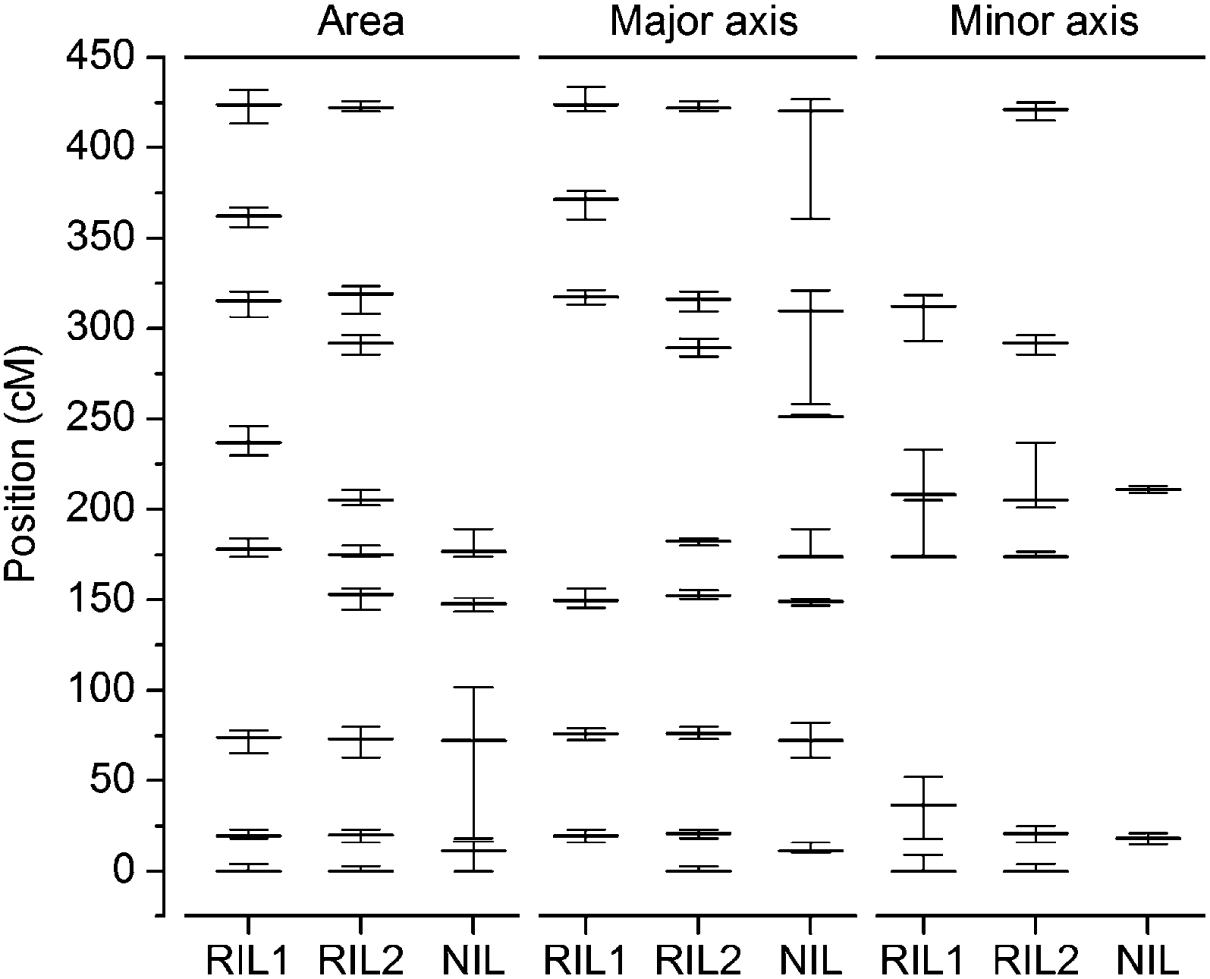
Confidence intervals of QTL identified by multiple-interval mapping for each indicated dataset. Regions were determined using a 1.5-LOD support interval, where the QTL is in the region in which the LOD score is within 1.5 of its maximum.

The RIL and NIL populations used here were previously genotyped with 234 and 102 markers, respectively, permitting QTL analysis of the three datasets using multiple interval mapping. Statistical significance of the QTL models was based on 25,000 permutations of the genotype against each phenotype, for each dataset (Churchill & Doerge 1994). LOD score profiles for each of the traits in each of the datasets are shown in Figure 3. Asterisks denote significant QTL. Allele effects associated with each QTL genotype are shown in Figure 4. The 1.5 LOD support intervals associated with each QTL are shown in Figure 5. The genomic position, associated marker, additive allele effect, and percentage of explained variance for each QTL are presented for the area trait (Table 2), major axis (Table 3), and minor axis (Table 4). Although the qtl library in R is not yet optimized for NIL populations, using an identical protocol as with the RIL population and interpreting the NIL results with caution allowed for ease of comparison between the three seed populations.

**Table 2:** QTL affecting seed area

Position	Associated Marker	Additive Effect, mm^2^	Variance Explained, %	LOD
RIL1				
1@0.0	PVV4	0.0043	4.6	4.1
1@19.6	GD.86L	0.0067	13.9	11.2
1@74.0	c1.loc74	0.0052	8.6	7.3
3@4.0	c3.loc4	−0.0041	7.4	6.4
3@63.0	c3.loc63	0.0036	4.5	4.0
4@64.0	c4.loc64	0.0060	12.2	10.0
5@36.0	c5.loc36	−0.0039	7.6	6.5
5@98.0	c5.loc98	0.0046	7.9	6.8
1@19.6:3@4.0	GD.86L: c3.loc4	0.0043	5.3	4.7
5@36.0:5@98.0	c5.loc36: c5.loc98	0.0020	1.5	1.4
RIL2				
1@0.0	PVV4	0.0054	8.5	7.6
1@20.0	c1.loc20	0.0048	6.5	5.8
1@73.0	c1.loc73	0.0033	3.9	3.6
2@42.7	Erecta	0.0043	7.0	6.3
3@1.0	c3.loc1	−0.0055	10.1	8.7
3@31.0	AD.92L	0.0033	3.6	3.4
4@40.6	DF.108L-Col	0.0041	6.3	5.7
4@67.8	GB.750C	0.0034	3.9	3.6
5@96.0	c5.loc96	0.0060	12.9	10.9
NIL				
1@11.2	m6	0.0077	21.0	9.1
1@72.2	c1.loc72	0.0051	8.4	4.1
2@37.3	m42	0.0051	11.8	5.6
3@2.9	c3.loc3	−0.0059	20.3	8.8
1@11.2: 1@72.2	m6: c1.loc72	0.0026	2.1	1.1

The genomic position, associated marker or pseudomarker, estimated additive effect of substitution of a Cvi allele at the indicated locus, and percentage of explained variance for each QTL present in the selected model with the highest LOD score. For RIL1, (T_f_, T_fv1_, T_i_, T_a_, T_av1_) = (5.51, 4.12, 3.40, 4.36, 2.63) and (T_m_, T_i_^H^, T_i_^L^) = (2.57, 3.40, 1.55). For RIL2, (T_f_, T_fv1_, T_i_, T_a_, T_av1_) = (5.50, 4.12, 3.42, 4.34, 2.58) and (T_m_, T_i_^H^, T_i_^L^) = (2.60, 3.42, 1.52). For NIL, (T_f_, T_fv1_, T_i_, T_a_, T_av1_) = (5.04, 3.51, 2.59, 4.55, 2.57) and (T_m_, T_i_^H^, T_i_^L^) = (2.74, 2.49, 0.76).

**Table 3:** QTL affecting seed length (major axis)

Position	Associated Marker	Additive Effect, mm	Variance Explained, %	LOD
RIL1				
1@19.6	GD.86L	0.011	15.3	10.6
1@76.0	c1.loc76	0.018	15.5	10.7
2@39.0	c2.loc39	0.012	8.1	6.0
4@66.0	c4.loc66	0.018	19.2	12.9
5@45.5	HH.480C	−0.0066	10.4	7.5
5@98.0	c5.loc98	0.011	6.5	4.9
1@19.6: 5@45.5	GD.86L: HH.480C	−0.011	6.6	5.0
RIL2				
1@0.0	PVV4	0.011	8.1	8.0
1@21.0	c1.loc21	0.013	10.5	10.0
1@76.3	GD.160C	0.0082	5.3	5.4
2@42.0	c2.loc42	0.016	21.3	17.9
3@8.3	CH.322C	−0.011	8.6	8.4
4@38.0	c4.loc38	0.0084	5.5	5.6
4@65.0	c4.loc65	0.013	11.8	11.0
5@96.0	c5.loc96	0.0099	8.3	8.1
1@21.0: 3@8.3	c1.loc21: CH.322C	0.0094	5.2	5.3
2@42.0: 5@96.0	c2.loc42: c5.loc96	0.0047	1.6	1.7
NIL				
1@11.3	c1.loc11	0.015	16.2	11.6
1@72.2	c1.loc72	0.014	17.8	12.4
2@38.5	c2.loc39	0.019	20.8	13.9
3@0.0	m53	−0.015	14.9	10.9
4@0.0	m69	−0.015	10.4	8.2
4@58.5	c4.loc59	0.023	10.4	8.2
5@95.2	c5.loc95	0.0095	4.6	4.0
4@0.0: 4@58.5	m69: c4.loc59	0.012	3.6	3.2

The genomic position, associated marker or pseudomarker, estimated additive effect of substitution of a Cvi allele at this locus, and percentage of explained variance for each QTL present in the selected model with the highest LOD score. For RIL1, (T_f_, T_fv1_, T_i_, T_a_, T_av1_) = (5.51, 4.12, 3.45, 4.33, 2.55) and (T_m_, T_i_^H^, T_i_^L^) = (2.59, 3.45, 1.53). For RIL2, (T_f_, T_fv1_, T_i_, T_a_, T_av1_) = (5.49, 4.09, 3.41, 4.34, 2.57) and (T_m_, T_i_^H^, T_i_^L^) = (2.58, 3.41, 1.51). For NIL, (T_f_, T_fv1_, T_i_, T_a_, T_av1_) = (4.65, 3.10, 2.16, 4.23, 2.20) and (T_m_, T_i_^H^, T_i_^L^) = (2.56, 2.16, 0.54).

**Table 4:** QTL affecting seed width (minor axis)

Position	Associated Marker	Additive Effect, mm	Variance Explained, %	LOD
RIL1				
1@0.0	PVV4	0.0077	9.4	5.0
1@36.6	AD.106L-Col	0.0068	7.3	3.9
3@0.0	DF.77C	−0.0068	7.1	3.8
3@34.0	c3.loc34	0.0063	6.1	3.3
4@61.0	c4.loc61	0.0068	7.5	4.0
RIL2				
1@0.0	PVV4	0.0077	10.9	8.3
1@21.0	c1.loc21	0.0072	9.0	7.0
3@0.0	DF.77C	−0.0082	14.5	10.7
3@31.0	AD.92L	0.0048	4.5	3.7
4@40.6	DF.108L-Col	0.0051	5.7	4.6
5@95.0	c5.loc95	0.0082	14.2	10.5
NIL				
1@18.3	c1.loc18	0.0091	22.2	6.3
3@37.1	c3.loc37	−0.0085	29.2	8.0

The genomic position, associated marker or pseudomarker, estimated additive effect of substitution of a Cvi allele at this locus, and percentage of explained variance for each QTL present in the selected model with the highest LOD score. For RIL1, (T_f_, T_fv1_, T_i_, T_a_, T_av1_) = (5.83, 4.58, 3.56, 4.49, 2.97) and (T_m_, T_i_^H^, T_i_^L^) = (2.55, 3.56, 2.03). For RIL2, (T_f_, T_fv1_, T_i_, T_a_, T_av1_) = (5.75, 4.50, 3.56, 4.47, 2.95) and (T_m_, T_i_^H^, T_i_^L^) = (2.55, 3.56, 1.95). For NIL, (T_f_, T_fv1_, T_i_, T_a_, T_av1_) = (7.80, 5.96, 4.63, 6.73, 4.89) and (T_m_, T_i_^H^, T_i_^L^) = (4.91, 4.63, 1.06).

For the area trait in RIL1, eight QTL were found to be significant (*P* < 0.001), including three on chromosome 1, two on chromosome 3, one on chromosome 4, and two on chromosome 5. The method of QTL identification used here is also capable of identifying interactions between loci, *i.e.*, epistatic relationships in which the effect on a trait of a genotype at one position influences the genotype effect at a second locus. Evidence of epistatic interactions between two pairs of loci was found in RIL1 for the area trait. They are indicated in Table 2 by a colon separating the labels of the two interacting loci. For example, a QTL at 19.6 cM on chromosome 1 interacted with a QTL at 4.0 cM on chromosome 3, or 1@19.6:3@4.0. For the major axis trait in RIL1, six QTL were chosen (*P* < 0.001), with one instance of epistasis detected (Table 3). QTL analysis of the minor axis trait in RIL1 revealed five significant loci (*P* < 0.001) with no evidence of epistasis, despite using a statistical approach (Manichaikul *et al.* 2009) that is relatively permissive of interactions in the selected model.

For RIL2, QTL analysis of seed area resulted in nine significant loci (*P* < 0.001), including three on chromosome 1, one on chromosome 2, two on chromosome 3, two on chromosome 4, and one on chromosome 5. No evidence of epistasis was found. For the major axis trait in RIL2, eight QTL were chosen (*P* < 0.001), with evidence of epistasis between two pairs of loci. For the minor axis trait, six QTL were identified (*P* < 0.001) with no evidence of epistasis.

For NIL, four significant loci were identified as contributing to the variation in seed area (*P* < 0.001), with two loci on chromosome 1, one locus on chromosome 2, and one locus on chromosome 3. Evidence of epistasis was found between the two loci on chromosome 1 (Table 2). Seven QTL were found to contribute to the variation in major axis in the NIL population (*P* < 0.001), with epistasis likely between the two loci on chromosome 4 (Table 3). For the minor axis trait, two QTL were identified (*P* < 0.001) with no evidence of epistasis.

**Figure 6:**
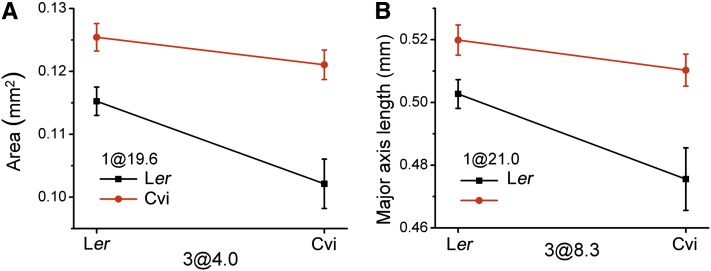
Genotype-phenotype plots for epistatic QTL pairs in the L*er* × Cvi population. Plotted points indicate two-locus genotype means ± SE for the two loci from (A) the area trait and (B) the major axis trait.

The plots in Figure 3 and Figure 5, and Tables 2−4 show that many of the associations between phenotype and genotype were repeatedly identified in the two independent RIL or NIL datasets, such as the area QTL on chromosome 1 in the 72.2−74.0 cM interval. In most but not all cases, Cvi DNA at the indentified positions had a positive effect on each of the traits relative to L*er* DNA. This is shown by the allele effect plots in Figure 4. Only one case of epistasis seems to have been detected twice, appearing in RIL1 and RIL2. The specifics of this apparently repeatable instance of epistasis are shown in Figure 6. Figure 6A shows a locus at position 4.0 on chromosome 3 to have an effect on the area trait that depended on the genotype at position 19.6 on chromosome 1. Figure 6B shows what appears to be the same locus, position 8.3 on chromosome 3, affecting the major axis trait to an extent that depends on the genotype at position 21 on chromosome 1.

## Discussion

Fertilization of the egg within the Arabidopsis ovule produces the zygote, and a second fusion event involving a different sperm and the polar nuclei triggers differentiation of endosperm tissue, which in turn provides nutrients for the developing embryo. At maturity, the endosperm will be reduced to a single cell layer, the remainder being absorbed by the cotyledons and axis of the embryo to fund seedling growth and development before the initiation of photosynthesis. The axis contains shoot and root meristematic tissue that give rise to all parts of the mature plant. In Arabidopsis, all of this is ultimately packaged within an oval seed half a millimeter in length. Because the embryo closely fills the seed (Figure 1), the results presented here may be viewed as genetic maps of embryo phenotypes.

### Benefits of the image processing technology

A feature of this study is the way in which automation made it feasible to include independent biological replicates of an RIL population and an NIL population without compromising sample size, resulting in more substantial evidence of multiple QTL than can be achieved by more typical phenotype analyses. The automation resulted by design from a good match between the capabilities of the custom analysis algorithm and the task of measuring the morphologies of 10^2^− 10^3^ individual, randomly scattered (not staged), sometimes-touching seeds captured in an image ranging from 100 to 300 MB in size. Input files were readily processed in automated batch form, generating results with higher resolution than previous studies achieved. In fact, allele effects on the phenotype as little as 5% were resolved.

In addition to seed area, the algorithm (available for download) measured seed length and width (major and minor axis, respectively). The multiple-QTL modeling of the measurements identified one locus unique to each of the two axis traits. A QTL on chromosome 2, position 41 was pertinent only to variation in the major axis, whereas one on chromosome 3, position 34 affected only the minor axis (Figure 5, Table 4). The major axis QTL on chromosome 2 was not detected by Alonso-Blanco *et al.* (1999) in their manual analysis of seed length, so it may be a dividend of the computational measurement method, or by Herridge *et al.* (2011), who used image analysis but with an algorithm that measured only seed area. Presumably, the major-axis effect on chromosome 2 was not large enough to affect area significantly or was compensated by an undetected reduction in minor axis. These comparisons with other studies and the internal comparisons made in Figure 5 indicate that area measurements capture most of the variation in length and width so that most seed size/shape QTL would be detected by measuring area only. However, by also measuring the two axes automatically, area effects could in most cases be ascribed to effects on width or length, or both (Figure 5).

### Differences in the two RIL populations

Although the two RIL populations were genetically identical, seeds in RIL2 were slightly larger than those in RIL1 (Figure 2). The RIL1 data set was obtained directly from seeds provided by another laboratory at the University of Wisconsin. The seeds for the RIL2 data set were obtained as described in the Materials and Methods. Differences in maternal environmental parameters, such as light quantity and watering schedules, may be responsible for the differences between RIL1 and RIL2 (Mousseau & Fox 1998; Elwell *et al.* 2011).

### Relation to previous seed size studies

Chromosome 1 position 0 and chromosome 1 position 19 were identified by Alonso-Blanco *et al.* (1999) as affecting seed length, and both also were found using the area phenotype in the two RIL and the NIL generations in this study. In addition, these loci were strongly supported using major and minor axis length as phenotypes in the three populations. An interval spanning these loci was also identified as an area QTL by Herridge *et al.* (2011). The QTL on the upper arm of chromosome 1 is thought to be the *MEDEA* locus. MEA, a FIS-class protein subunit of the *Polycomb*-group complex, is a chromatin modifying enzyme that acts in the *FIS* group of genes that mediate seed development by repressing expression of target genes (Chaudhury *et al.* 2001; Köhler & Grossniklaus 2002; Köhler *et al.* 2003). Before fertilization, gene expression is only from the maternal allele, although the paternal allele functions after fertilization (Grossniklaus *et al.* 1998; Kinoshita *et al.* 1999; Luo *et al.* 2000; Yadegari *et al.* 2000). *FIS* genes are thought to be negative regulators of endosperm growth and development. *fis* mutants undergo seed development without fertilization, and the endosperm does not cellularize but enlarges during the later stages of seed growth (Chaudhury *et al.* 1997; Kiyosue *et al.* 1999). The locus at the top of chromosome 3 was identified in the present study as well as by Alonso-Blanco *et al.* (1999) but no information supporting a candidate gene could be found.

Both length loci found by Alonso-Blanco *et al.* (1999) on chromosome 4 also were found to affect major axis in this study. One of these loci has previously been predicted to be *APETALA2* (*AP2*) (Jofuku *et al.* 2005). However, like Herridge *et al.* (2011), our methods resulted in a smaller confidence interval that excludes *APETALA* but could colocalize with *SHORT HYPOCOTYLS UNDER BLUE1* (*SHB1*). Lack of AP2 activity has been shown to impact seed development. Mutants in this gene are irregularly shaped and have increased amounts of seed proteins and fatty acids (Jofuku *et al.* 1994; Leon-Kloosterziel *et al.* 1994; Western *et al.* 2001). SHB1 has been shown to associate with the promoter regions of *MINI3* and *IKU2*, whose mutants show a reduced seed size (Luo *et al.* 2005; Zhou *et al.* 2009; Wang *et al.* 2010). All three of these genes have been implicated in controlling the timing of endosperm growth and cellularization (Zhou *et al.* 2009).

Of the two QTL on chromosome 5 found in the Alonso-Blanco study, both also were found in at least one of populations analyzed here, with stronger evidence for the locus near the bottom of the chromosome. Three genes with known roles in seed size development colocalize with this QTL: *ARF2*, *TITAN3*, and *AGL62*. *ARF2* encodes a transcription factor that binds auxin-responsive elements in auxin-regulated genes’ promoter regions (Ulmasov *et al.* 1999; Schruff *et al.* 2006). Its role in cell proliferation was revealed as the integument of a mutant plant contains supernumerary cells without previous fertilization of the ovules (Schruff *et al.* 2006). *TITAN3* has been shown to be involved with the proliferation of endosperm nuclei early in seed development, although its mutants have mostly normal embryo development and undergo cellularization of the endosperm at the proper time (Liu & Meinke 1998). *AGL62* helps to generate a mobile signal to initiate seed coat development, probably through interaction with type I MADS-box proteins such as PHERES1 (de Folter *et al.* 2005; Roszak & Kohler 2011).

The minor axis QTL on chromosome 3 appears to match the seed area QTL identified by Herridge *et al.* (2011) in a Bur × Col RIL population but, appropriately, it was not found by Alonso-Blanco *et al.* (1999) in their study of length. Figure 4 shows that substitution of Cvi alleles at this minor-axis locus had a positive effect on the phenotype in the RIL populations, but a negative effect in the NIL population. This disagreement could be due to two loci with opposite effects acting within the confidence interval found in the RIL population, whereas only one of these loci was able to be identified in the NIL population, possibly due to the smaller amount of genetic recombination in these lines. Alternatively, this locus could act epistatically with another locus, but again lack of recombination in NIL could prevent the same results from being witnessed in this data set. These data indicate strong support for this locus acting to control the width of the seed, without an impact on the length.

QTL that affect the major axis may identify loci that contribute to the starting length of the embryo, and some of those may play a general role in regulating plant size. The QTL on chromosome 2 contributing to variation in seed area and length but not width could, based on its position, be the *ERECTA* locus that is nonfunctional in the L*er* parent of the population used here (Koornneef *et al.* 2004). The *ERECTA* locus, which is responsible for the smaller stature of the adult in the Landsberg *erecta* accession, also was found in a QTL analysis of developmental traits using the Col x L*er* RIL population (Kearsey *et al.* 2003) and various correlated growth traits in a Kas × L*er* population (Prinzenberg *et al.* 2010). Although the Cvi accession has relatively large seeds, its fresh weight and leaf length and width are consistently smaller than those of other accessions (Stokes *et al.* 2007).

### Relation to previous studies of plant biomass

Several studies have found strong correlations between metabolic activity and biomass (Cross *et al.* 2006; Meyer *et al.* 2007; Lisec *et al.* 2008; Steinfath *et al.* 2010) so QTL that are involved in the variation in seed size could be due to metabolic activity within the seed influencing the size of the axis or the cotyledons. Calenge *et al.* (2006), researching the QTL associated with carbohydrate metabolism in different nitrogen environments, found approximately 14 distinct loci that affect sugar concentration in the Bay-0 × Shahdara RIL population. One locus identified in an analysis of starch content in a nitrogen-rich environment, ST10.2, colocalizes with one of the QTL identified here on the distal end of chromosome 1. Another locus associated with starch and fructose content maps to the same region as a QTL identified here on the proximal end of chromosome 3.

### Relation to previous studies of seed content QTL

Seed oils are an important part of human and animal nutrition and also have uses in industrial applications. In many species, they are the fuel for the germinating seedling that enable establishment. Triacylglycerols are the main storage component for seed oils in many species, and Arabidopsis provides an excellent model system for the commercially relevant *Brassica* oilseed crops due to its similar seed physiology and subsequent development. Arabidopsis, whose oil content is approximately 40% of seed dry weight, has successfully been used to probe natural variation of fatty acid content in seeds (Lemieux *et al.* 1990; Millar & Kunst 1999; O’Neill *et al.* 2003). The sheer bulk of oil within the Arabidopsis seed makes it likely that some loci involved in variation in seed size may also be involved in controlling fatty acid content, although one study found no strong link between seed oil content and seed mass (Hobbs *et al.* 2004).

Indeed, two of the loci identified in our study colocalize with QTL involved in fatty acid content. A locus near the end of chromosome 2 is in the same region as a locus that accounts for 17% of the variation in seed oil content in the L*er* × Cvi RIL population (Hobbs *et al.* 2004), and could be due to the action of *FAD3*, which encodes a fatty acid desaturase present in the endoplasmic reticulum. Overexpression of FAD3 has been shown to decrease linoleic acid and increase linolenic acid content (Shah *et al.* 1997). Mutations at this locus are semidominant, supporting the idea that this gene could contribute to variation in seed oil phenotypes. Further support for this locus was found in research using numerous RIL populations from diverse environments (O’Neill *et al.* 2012). However, this locus is also near that of *ERECTA*, and since our analyses only identified this region when using area and major axis, but not minor, as phenotypes, we believe it is probably *ERECTA* that is instead the underlying gene behind this QTL.

Another locus that has been identified as being involved in the control of seed oil content is that of *FAE1*, a 3-ketoacyl-Coa synthase that works to synthesis very-long-chain fatty acids in the endoplasmic reticulum of cells within the developing embryo. The proportions of very-long-chain fatty acids in seed oil have been found to be quantitatively affected by transcription level of *FAE1* (Millar & Kunst 1997), and this locus has been identified in QTL analyses of seed oil quantity (O’Neill *et al.* 2003, 2012). *FAE1* is localized to the distal end of chromosome 4, where our study has consistently identified a region that contributes to seed size variation. If relationships between seed oil and some morphological feature detectable by image analysis can be established, the approach to phenotyping used here could be used to select genotypes with desirable chemical compositions.
